# C5aR1 and cGAS/STING and their possible involvement in radiosensitivity of colorectal cancer

**DOI:** 10.1016/j.isci.2026.115009

**Published:** 2026-02-12

**Authors:** Quoc Thao Trang Pham, Pei-Ju Lee, Nguyen Quoc Khanh Le

**Affiliations:** 1 Graduate Institute of Clinical Medicine, College of Medicine, Taipei Medical University, Taipei 110301, Taiwan; 2 International Ph.D. Program in Cell Therapy and Regenerative Medicine, College of Medicine, Taipei Medical University, Taipei 110301, Taiwan; 3 Department of Dermatology, Faculty of Medicine, University of Medicine and Pharmacy at Ho Chi Minh City, Ho Chi Minh City 700000, Vietnam; 4 Graduate Institute of Medical Sciences, College of Medicine, Taipei Medical University, Taipei 110301, Taiwan; 5 Department of Oncology, University of Oxford, Oxford, UK; 6 In-Service Master Program in Artificial Intelligence in Medicine, College of Medicine, Taipei Medical University, Taipei 110301, Taiwan; 7 AIBioMed Research Group, Taipei Medical University, Taipei 110301, Taiwan; 8 Translational Imaging Research Center, Taipei Medical University Hospital, Taipei 110301, Taiwan

**Keywords:** Immune system, Cancer

## Abstract

Worldwide, colorectal cancer (CRC) stands as the third leading cancer in terms of both diagnosis and mortality, underscoring its significant global health impact. Enhancing radiotherapy with radiosensitizers, such as C5aR1 (complement C5a receptor 1) blockade, has shown promise in treating resistant CRC, particularly in immunologically cold tumors. However, the molecular mechanisms by which C5aR1 inhibition improves radiosensitivity remain to be clarified. Reduced cGAS/STING (cyclic guanosine monophosphate-adenosine monophosphate synthase/stimulator of interferon genes) pathway activity is linked to radioresistance, while radiotherapy-induced cGAS/STING activation increases IFNB1 (interferon beta 1) and impairs DNA repair. Conversely, the complement component 5a (C5a)/C5aR1 axis suppresses STING-driven IFNB1 expression in immune responses, suggesting their distinct regulatory effects on cancer cell radiotherapy responses. Targeting C5aR1 may therefore enhance radiosensitivity by modulating the cGAS/STING pathway. This review examines potential interactions between C5aR1 and the cGAS/STING pathway, highlighting their relevance to addressing resistance mechanisms in CRC.

## Graphical abstract

**Figure:**
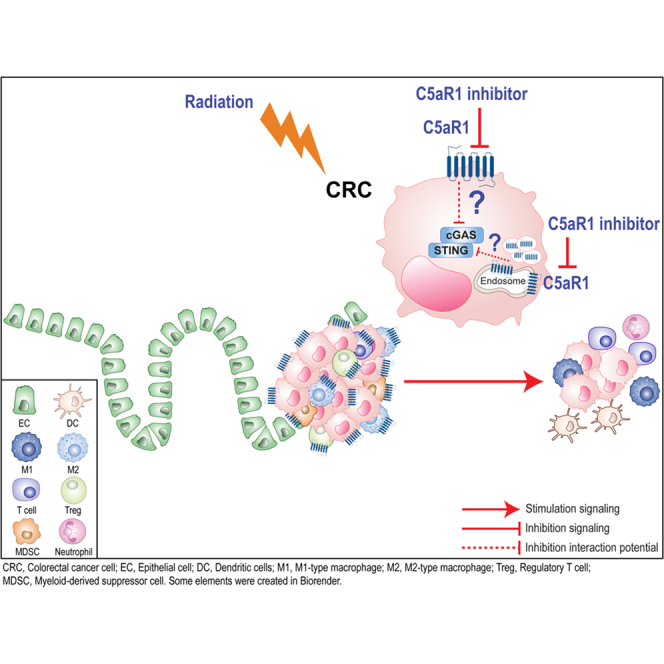

## Introduction

Globally, colorectal cancer (CRC) is among the top three cancers in terms of incidence, and approximately 65% of patients receive their diagnosis at an advanced or metastatic stage. The 5-year survival rate for these individuals is a mere 15%.^1^ While surgery is the primary treatment, only around 32.9% of patients have their tumors detected at an early stage and are localized.^2^ Typically, most CRC patients, identified at advanced stages, require local radiotherapy (RT) as an adjunctive treatment.^3^ However, RT often falls short in CRC, possibly because tumor cells acquire radioresistance, diminishing treatment efficacy and hindering optimal therapeutic outcomes.^4^ RT resistance results from tumor-intrinsic properties and complex immune-mediated mechanisms, underscoring the need to dissect both dimensions in greater depth to improve RT-based treatments and diminish the risk of therapeutic failure. Targeting radiosensitivity, especially in immunosuppressive tumors, could boost treatment success in the most difficult-to-treat cases.

RT not only delivers local treatment to CRC cells but also induces dramatic changes in the tumor microenvironment (TME).^5^ RT is administered to over half of CRC patients as an integral part of their treatment plan. RT primarily causes permanent DNA damage in rapidly proliferating cells, especially by inducing lethal double-strand breaks (DSBs).^6^ Disruption of DNA repair circuitry in CRC cells can tip the balance between survival and death after irradiation, altering radiosensitivity and fostering radioresistance.^7^^,^^8^^,^^9^ Simultaneously, exposure to radiation can influence both innate and adaptive immune responses. It does so by enhancing the expression of major histocompatibility complex 1 (MHC-1) molecules on tumor cells and initiating cell death pathways that trigger immune activation.^10^^,^^11^^,^^12^ As a result, RT accomplishes two key outcomes: it eradicates malignant cells and simultaneously transforms the TME, encouraging conditions that enhance anti-tumor immune activity.

Clinical outcomes following RT in CRC remain modest, as disease stabilization is observed in approximately one-quarter of patients and progression-free survival generally ranges between 1.8 and 2.5 months, potentially due to limited tumor responsiveness to radiation.^13^ This may be associated with the immunosuppressive TME, which is a key contributor to radioresistance.^14^ RT can strengthen tumor-directed immune responses through several pathways. It can promote the entry of CD8^+^ cytotoxic T cells into the tumor bed, increase the production of interferon beta 1 (IFNB1), enhance antigen cross-presentation, and drive the maturation of intratumoral dendritic cells (DCs).^15^^,^^16^ However, these beneficial effects are blunted in an immunosuppressive TME, characterized by exhausted regulatory T cells (Tregs) and a paucity of macrophages, DCs, and CD8^+^ T cells.^17^ In CRC, the various cellular components of the TME also differ in their intrinsic sensitivity to radiation. Endothelial cells and DCs tend to be highly radioresistant, while TAMs (tumor-associated macrophages) and cancer-associated fibroblasts are also relatively resistant. By contrast, monocytes and lymphocytes are much more susceptible to radiation-induced damage.^18^ These characteristics of the TME make the response of CRC patients to RT more complex. Emerging data point to specific, radiation-responsive signaling hubs as key architects of immune suppression in CRC. The progression of CRC is significantly affected by two immune pathways: the complement component 5a (C5a)/complement component 5a receptor 1 (C5aR1) signaling axis and the cyclic guanosine monophosphate-adenosine monophosphate synthase (cGAS)/stimulator of IFN genes (STING) pathway, both of which play essential roles in tumor-immune interactions. Although these pathways are triggered by RT, they contribute to sustaining an immunosuppressive TME. These molecular conduits, by entrenching immune evasion, may paradoxically blunt radiotherapeutic outcomes, thus unveiling themselves as strategic levers to recalibrate radiosensitivity in refractory CRC.

## C5aR1 and cGAS/STING in CRC response to radiation therapy

### Complement pathway C5a and its receptor C5aR1 in RT

#### Role of C5a/C5aR1 in the RT response

Local RT activates the complement system systemically by triggering C5 (complement component 5) and C3 (complement component 3), which leads to apoptosis, mitotic catastrophe, and necrosis.^19^^,^^20^ Among the first innate immune responses activated after RT is the complement pathway, particularly C5a/C5aR1.^21^ Proteolytic processing of the C5 complement component is triggered upon activation, releasing the C5a fragment. This fragment then binds to its receptors, initiating the formation of downstream effectors.^22^ C5a exerts its biological imprint predominantly via engagement with C5aR1, a G-protein-coupled receptor (GPCR). Upon ligation, this receptor choreographs a diversified signaling relay, mobilizing heterotrimeric G proteins, namely G16, Gi, and Gq, in concert with β-arrestins, thus orchestrating a multifaceted intracellular cascade. These interactions then initiate several cellular responses, such as cytokine secretion, cell movement, and phagocytosis.^22^ Notably, C5a also interacts with C5aR2, an atypical GPCR that, unlike C5aR1, does not exhibit detectable functional coupling with G proteins. Instead, C5aR2 primarily recruits β-arrestins, imparting unique immunomodulatory properties that often suppress the inflammatory response.^23^^,^^24^^,^^25^ C5aR1 coordinates immune responses in CRC through its unique ability to engage both G protein subtypes and β-arrestins, acting as a molecular switch that shifts between G-protein-dependent and independent signaling pathways. For example, β-arrestins typically mediate receptor downregulation and endocytosis, effectively terminating G-protein-dependent signaling.^26^ However, β-arrestins can either independently activate downstream signaling pathways or collaborate with G proteins^26^ to enhance chemotaxis and cytokine secretion.^27^

RT not only eliminates malignant cells through direct cytotoxicity but also triggers a short-lived burst of complement activity within the TME. RT triggers activation of the complement cascade in both preclinical mouse tumor and human cancer specimens, swiftly driving the production of the anaphylatoxins C3a and C5a.^19^ C3a and C5a serve as pivotal molecular enhancers, linking the cell death triggered by radiation to the onset of tumor-specific immune responses, thereby embedding immune activation as a fundamental aspect of the therapeutic outcome post-irradiation.^19^ Blocking the receptors for C3a (C3aR) or C5a (C5aR1) reduced the immune-mediated tumor response to radiation.^20^ This may reflect C5aR1 and C3aR1 signaling, enhancing DC maturation, boosting CD4^+^-T-cell-derived IFN-γ,^28^ and limiting FoxP3^+^ (Forkhead box P3) Treg development,^29^ which contribute to RT efficacy. Furthermore, radiation treatment prompted an upregulation of C3aR1 and C5aR1 on DCs residing within the tumor.^19^ Effective dendritic cell maturation is required to maintain cytotoxic T cell activity and support optimal RT responses.^15^ Ablation of C3aR1 and C5aR1 signaling compromises IFN-γ release from intratumoral CD8^+^ T cells and promotes Treg accumulation,^19^ potentially favoring transforming growth factor β1 (TGF-β1)-driven FoxP3^+^ Treg differentiation over pro-inflammatory CD4^+^ effector T cells.^29^

Although complement activation can support the tumor-restraining actions of RT, the C5a and its receptor C5aR1 have been linked to CRC cells’ resistance to irradiation. In CRC, heightened complement activity is associated with an inflammatory milieu, unfavorable prognosis, and strong complement-related transcriptional signatures.^30^^,^^31^^,^^32^ Intracellular C5a/C5aR1 signaling can promote CRC tumorigenesis by stabilizing β-catenin.^33^ Engagement of C5aR1 on TAMs by the anaphylatoxin C5a induces NF-κB (nuclear factor κB)-dependent polarization toward an immunosuppressive, M2-like phenotype, thereby facilitating CRC metastatic progression.^34^ Moreover, some preclinical studies have demonstrated that blocking C5aR1 enhances anti-tumor immunity.^21^^,^^35^^,^^36^^,^^37^ A previous study identified C5aR1 in the complement system as a druggable target with the potential to improve CRC treatment. In CRC, poorer overall survival is strongly associated with higher C5aR1 expression.^21^ Notably, pharmacological inhibition of C5aR1 using the antagonist PMX205 enhanced radiation responses and reduced CRC cell survival *in vivo*,^21^ particularly in addressing radioresistance. The activation of C5a/C5aR1 was notably higher in radioresistant SW837, SW1463, and HRA-19 cell lines than in radiosensitive HCT116 cells.^38^ CFB (complement factor B) was upregulated in radioresistant cell lines, consistent with activation of the alternative complement pathway.^39^ In addition, CFB levels showed a positive association with overall C5 expression in these cells, suggesting coordinated regulation of alternative-pathway components.^39^ In radioresistant CRC, the major contributors include an immunosuppressive TME and intracellular compensatory pathways in cancer cells that sustain tumor survival.^14^^,^^17^^,^^40^^,^^41^^,^^42^ Reportedly, inactive C5aR1 signaling has been reported to augment RT efficacy in settings lacking CD8^+^ T cell infiltration, while elevated C5aR1 expression in tumor tissue correlates with diminished radiation responsiveness^21^^,^^35^^,^^36^^,^^37^ and enhanced intracellular alternative pathways that preserve tumor survival.^33^^,^^43^^,^^44^ Collectively, C5aR1 exerts two opposing influences on CRC during RT: it heightens radiosensitivity by activating immune players such as DCs and cytotoxic T cells, yet it simultaneously reinforces radioresistance within tumor cells by driving growth, survival, and metastatic programs. Thus, blocking C5aR1 can either restrain or promote tumor growth following RT, with the net effect determined by which C5aR1-expressing cell populations (e.g., malignant epithelial cells vs. immune cells) and which types of C5a/C5aR1 downstream responses (canonical vs. noncanonical signaling) dominate the radiation-induced response. Overall, disrupting C5a/C5aR1 signaling represents a promising strategy to counter CRC radioresistance.

#### Inhibition of C5aR1 to improve CRC treatment outcomes

C5aR1 enrichment in colorectal tumors aligns with inferior treatment outcomes. A plausible mechanism is that tumor-derived C5a engages C5aR1 on myeloid-derived suppressor cells (MDSCs), promoting their buildup within the tumor compartment. This MDSC accumulation dampens CD8^+^ T cell function and reinforces an immunosuppressive microenvironment conducive to tumor expansion.^35^ Consistent with these findings, RT-induced activation of C5a signaling in colorectal tumors correlates with increased recruitment of MDSCs, attenuated cytotoxic T cell responses, and amplification of tumor-driving inflammatory signals.^35^^,^^45^ C5aR1 is also present on other immune cells, including macrophages, neutrophils, and DCs, where its signaling helps shape a pro-inflammatory milieu that favors metastatic spread.^46^^,^^47^ In addition, engagement of C5aR1 by C5a promotes the dissemination of CRC by driving TAMs toward an M2-like, metastasis-supportive phenotype.^34^ Taken together, inhibiting C5aR1 is expected to alleviate immunosuppressive TME and bolster anti-tumor responses, a concept supported by findings in other cancers, including squamous malignancies and breast tumors.^48^^,^^49^ Yet, one investigation in CRC reported that combining C5aR1 blockade with RT improved therapeutic outcomes without a marked rise in cytotoxic T cell infiltration; instead, the benefit correlated with reduced C5aR1 levels in tumor tissue and enhanced NF-κB-mediated apoptosis.^21^ Adding to this complexity, aberrant expression of C5aR1 on CRC cells has been shown to increase their motility and invasive potential.^50^ Thus, the functional impact of C5aR1 in CRC reflects its dual presence on both immune populations and the cancer cells themselves.

**Figure 1:**
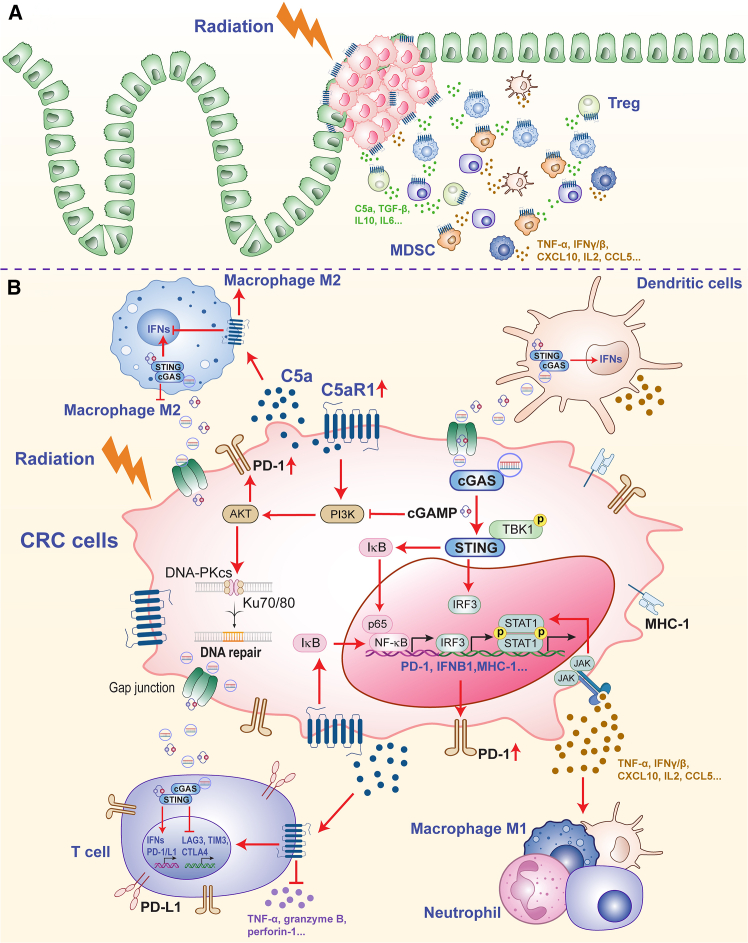
The attributes of radioresistance associated with C5aR1 and the cGAS/STING pathway in colorectal cancer (A) The immunosuppressive tumor microenvironment (TME) persists following radiotherapy in CRC. C5a/C5aR1 expression has been observed to increase after radiation, leading to the recruitment of immunosuppressive components such as regulatory T cells, myeloid-derived suppressor cells, and tumor-associated macrophages. Concurrently, radiation-therapy-driven activation of cGAS/STING elicits a surge of pro-inflammatory cytokines and chemokines (IL-2, CXCL10, CCL5, IFN-γ/β, and TNF-α) and reinforces granzyme-B-mediated cytotoxicity, thereby opposing the tolerogenic conditions imposed by strong C5a/C5aR1 signaling. The latter enhances IL-10, TGF-β, and IL-6 while suppressing granzyme B, IFN-γ, perforin-1, and inflammatory mediators such as IL-18, IL-12, IL-2, and CXCL9. (B) The contrasting functions of C5aR1 and cGAS/STING highlight their potential interaction in modulating resistance to radiotherapy in CRC. In CRC, C5a/C5aR1 signaling in immune cells promotes an immunosuppressive tumor microenvironment by recruiting myeloid-derived suppressor cells, impairing CD8^+^ T cell activity, and supporting protumor, M2-like tumor-associated macrophages, and increased immune checkpoint expression (including PD-1/PD-L1). High C5aR1 expression on CRC tumor cells and macrophages is linked to poor prognosis and resistance to immunotherapy, and its inhibition can repolarize macrophages toward an M1 phenotype and enhance treatment responses, partly via PI3K/AKT- and NF-κB-related pathways, which are also associated with cGAS/STING pathway activation. In particular, cGAMP, a co-activator of STING, has been shown to overcome chemoresistance in CRC by inhibiting the PI3K/AKT/mTOR pathway, an axis also implicated in radioresistance due to its role in promoting DNA repair. cGAS/STING activation has also been linked to adaptive upregulation of PD-1, highlighting the complex and context-dependent interplay between innate immune activation and immune regulation. CRC, colorectal cancer cell; Treg, regulatory T cell; MDSC, myeloid-derived suppressor cell; TNF, tumor necrosis factor; IFN, interferon; CXCL, C-X-C motif chemokine ligand; CCL, C-C motif chemokine ligand; IL, interleukin; C5a; complement component 5a; C5aR1, complement component 5a receptor 1; cGAS, cyclic GMP-AMP synthase; STING, stimulator of interferon genes; TBK1, TANK-binding kinase 1; NF-κB, Nuclear factor κB; IRF3, interferon regulatory factor 3; PD-1, programmed cell death protein 1; PD-L1, programmed death-ligand 1; cGAMP, cyclic GMP-AMP; IκB, inhibitor of kappa B; PI3K, phosphoinositide 3-kinase; JAK, Janus kinase; STAT1, signal transducer and activator of transcription 1; MHC-1, major histocompatibility complex class I; DNA-PKCs, DNA-dependent protein kinase complexs; IFNB1, interferon beta 1; LAG3, lymphocyte-activation gene 3; TIM3, T cell immunoglobulin and mucin domain-containing protein 3; CTLA4, cytotoxic T-lymphocyte antigen 4. Some graphical elements were created using BioRender (Pham, Q. (2026) https://BioRender.com/tf61v6z).

Although elevated levels of C5aR1 within CRC tissue have been documented, its precise distribution in CRC cells and the mechanisms by which these pools influence disease progression remain insufficiently defined. Using both chronic-colitis-driven and Apc-deficient mouse models, together with human CRC specimens, Ding et al. identified a cytoplasmic reservoir of C5aR1 in CRC cells. This intracellular fraction was shown to foster tumor development by supporting β-catenin stabilization, implying that targeting this specific source of C5aR1 could impede colorectal tumorigenesis.^33^ In addition to sustaining β-catenin stability, intracellular C5a/C5aR1 activity correlates with enhanced transcription of β-catenin-responsive genes, notably *CCND1* (*Cyclin D1*) and *SURVIVIN*, while sustaining the abundance and functional integrity of proteins associated with *CTNNB1* (*catenin beta 1*), the gene that encodes β-catenin.^33^ These findings further support the emerging view that complement components, once regarded as acting predominantly at the cell surface or within the intracellular space, also exert critical regulatory functions inside cancer cells. Additionally, the authors applied PMX205 at varying concentrations to target cytoplasmic C5aR1. Dose-dependent blockade of C5aR1 curtailed azoxymethane (AOM)/dextran sodium sulfate (DSS)-driven colorectal tumor formation and was accompanied by reduced β-catenin protein abundance in the chronic-colitis-associated CRC model, with the strongest effects observed at the highest PMX205 dose.^33^ Notably, this family of cyclic peptides exhibits very limited intracellular permeability, suggesting that their principal action is restricted to C5aR1 located on the cell surface.^51^ These findings raise important questions for future investigation, particularly regarding whether PMX205 can alter intracellular pools of C5aR1 in a dose-dependent manner. It should further emphasize the need to assess other, more cell-permeable C5aR1 blockers, such as JPE-1375 or Avacopan,^52^^,^^53^^,^^54^ which could provide more efficient targeting of cytoplasmic C5aR1 in CRC cells. This issue becomes especially relevant under hypoxic conditions, where C5aR1 is driven into the intracellular compartment, potentially diminishing the effectiveness of therapies aimed solely at surface-expressed C5aR1. Under hypoxic stress, CRC cells activate an unfolded protein response (UPR) that drives the progressive accumulation of endocytosed C5aR1 in intracellular compartments (Figure 1).^54^ This hypoxia-induced, UPR-dependent redistribution of C5aR1 suppresses both autophagy and apoptosis, thereby enhancing the survival capacity of tumor cells in low-oxygen conditions.^54^ Consistent with the idea that hypoxia drives C5aR1 into intracellular reservoirs, the strongest survival-modulating effects are seen with highly cell-permeable C5aR1 antagonists, such as Avacopan.^54^ Together, emerging evidence is reshaping our understanding of how C5aR1 is regulated and functions within CRC cells. A key remaining question is how the relative contributions of membrane-bound versus intracellular C5aR1 pools influence CRC initiation and subsequent growth, a topic that warrants thorough investigation.

Agents that block C5aR1 are gaining attention as potential therapies to enhance the effectiveness of CRC treatment. C5aR1 inhibition has been shown to effectively suppress colorectal tumorigenesis^35^ and improve the RT response, including in immunosuppressive tumors, without affecting normal tissues.^21^ Long-standing inflammation and injury to the intestinal lining, seen in conditions like inflammatory bowel disease (IBD), substantially increase the likelihood of developing CRC. These settings are often characterized by elevated levels of complement proteins, particularly C5a. Administration of the C5aR1 inhibitor PMX205 in the DSS colitis mouse model markedly reduced colonic tissue inflammation and overall disease burden. This therapeutic effect coincided with a decline in pro-inflammatory mediators (interleukin-6 [IL-6], IL-1β, and tumor necrosis factor [TNF]) and elevated levels of the anti-inflammatory cytokines IL-10 and IL-4, indicating that targeting C5aR1 can dampen complement-driven inflammation that may promote CRC development.^55^ Furthermore, PMX205 sharply curtailed MDSC accumulation within colon tissue, while enriching macrophages and CD8^+^ T cells within the TME, collectively restraining CRC growth.^35^ Additionally, preclinical studies indicate that PMX205 can protect against radiation-induced cognitive impairments in mice, including those with glioblastoma, by reducing neuroinflammation and preserving synaptic integrity, without interfering with the tumor-suppressing effects of RT.^56^ Collectively, these observations point to PMX205 as a candidate for reducing radiation-related neurological side effects,^57^ yet still preserving RT’s therapeutic impact on tumors.

C5aR1 inhibitors have shown promising results in alleviating inflammation while maintaining the essential protective roles of the complement system. Following intravenous administration, both C5aR1 antagonists, PMX205 and PMX53, are rapidly distributed and eliminated, primarily via renal excretion. PMX205 has been shown to be well tolerated in long-term studies and repeated dosing, with no evidence of drug accumulation following daily administration.^51^^,^^58^ Moreover, blocking C5aR1 does not appear to interfere with vital complement system activities, including opsonization (driven by C3b) or the destruction of target cells by the membrane attack complex (MAC; C5b–9).^59^ In models of meningococcal sepsis, either removing C5aR1 genetically or inhibiting it pharmacologically with PMX53 or PMX205 enhanced survival and dampened cytokine responses, yet the ability to clear bacteria remained unaffected.^60^ This likely reflects that complement-dependent bacterial killing, including MAC activity, remains operational despite the interruption of C5a/C5aR1 signaling. As a result, the C5aR1 inhibitor shows strong potential as a therapeutic adjunct to RT in CRC (Table 1). Yet, the specific pathways and radiosensitizing effects of C5aR1 inhibitors in CRC remain unclear, emphasizing the need for further research.

**Table 1:** Preclinical studies of C5aR1 inhibitors and cGAS/STING activators in CRC treatment

Target	Combined treatment		Animal model	Cell lines		Reference
C5AR1	PMX205	RT (fractionated RT [3 × 4.45 Gy] or single-dose RT [9 Gy])	AKPT (Apc [Apc^fl/fl^], the oncogene Kras [Kras^G12D/+^], the tumor suppressor Trp53 [p53 ^fl/fl^], and the TGF-β pathway [Alk5/Tgfbr1^fl/fl^]) organoid culture MC38 tumor-bearing mice	human HCT116, HT29 murine MC38	↓ tumor growth (tumor volume) ↓ tumor cell death ↑ radiosensitivity	Beach et al.^21^
		–	AOM/DSS mouse model	–	↓ CRC tumorigenesis ↓ MDSCs ↓ proinflammatory cytokines TNF-α, IL-1α, IL-6, IL-1β, IL-17A, IL-11 and the chemokines CCL2, CCL17, CXCL1, and CXCL5 ↑ CD8^+^ T cells, macrophages ↑ anti-inflammatory cytokine (IL-23, IL-9, IL-27, and especially IL-10)	Ding et al.^35^
		–	AOM/DSS mouse model	human HCT 116, SW620 murine CT26	↓ CRC tumorigenesis ↓ β-catenin, cyclin D1, and COX-2	Ding et al.^33^
	PMX-53	–	MC38 tumor-bearing mice	–	↓ tumor growth (tumor size and tumor weight) ↑ CD8^+^ T cells and M1 macrophages	Zhao et al.^37^
cGAS/STING signaling	CD73 inhibitors^a^	radiotherapy (an 8-Gy irradiation)	MC38-OVA tumor-bearing mice	murine CT26, MC38	↓ tumor growth (tumor volume) ↑ survival rate ↑ IFN-γ, TNF-α, CD8^+^ T cells function ↓ PD-1, TIM3, TAMs, MDSCs, Tregs	An et al.^61^
	TLC388 (topoisomerase I inhibitor)^a^	radiotherapy (5Gy) anti-PD-1	CT26 tumor-bearing mice	human SW480, SW620, HCT116, HT29, CoLo320DM murine CT26	↓ tumor growth (tumor volume) ↓ tumor cell death ↑ CD4^+^ and CD8^+^ T cells, NK cells, DCs ↑ IFN-β1, CXCL10, IL-12A, TNF-α, and granzyme B ↑ anti-PD-1 efficacy	Chen et al.^62^
	AZD0156 (a selective oral ATM inhibitor)^a^	radiotherapy (8 Gy × 2)	–	human HCT116, SW480, SW620 murine CT26, MC38	↓ tumor growth (tumor volume) ↑ radiosensitization, anti-PD-L1 ↑ CD8^+^ T cells, CXCL10, CXCL11, IFNB1, CTL-1, CTL-2	Xie et al.^63^
	PREX-in1 (a PREX2 small-molecule inhibitor)^a^	radiotherapy (8 Gy × 3 fractions)	MC38, CMT93, and IR-CMT93 tumor-bearing mice	human SW480, CaCo2, HCT15, HCT116, SW620, HT29, LoVo, RKO murine MC38	↓ tumor growth (tumor volume, tumor weight) ↑ CD8^+^ T cells ↑ radiosensitivity	Li et al.^64^
	berzosertib (ATR inhibitor)^a^	radiotherapy (a single fraction of 5 Gy X-ray dose) anti-PD-L1	MC38, CT26 tumor-bearing mice	human HCT116, SW480 murine CT26, MC38	↑ anti-PD-L1 efficacy ↑ CD8^+^ T cell infiltration ↑ type I interferon signaling, NF-κB, and TRAF6-mediated IRF7	Liu et al.^65^
	irinotecan (IRIN)^a^	radiotherapy (2 Gy X-rays for 4 fractions) anti-PD-1	MC38 tumor-bearing mice	human HCT8, HT29 murine MC38	↑ radiosensitivity ↑ tumor cell death ↑ CD4^+^ and CD8^+^ T cells, and DCs ↑ IFN-α, IFN-β, PD-L1, MHC-1 ↑ anti-PD-1 efficacy	Wang et al.^66^
	midostaurin (PKC412)^a^	anti-PD-1	CT26 tumor-bearing mice	human HCT116, SW480	↓ tumor growth (tumor volume) ↓ Tregs ↓ c-KIT, Flt3, Trex-1 ↑ IFN-β, M1 macrophage ↑ anti-PD-1 efficacy	Lai et al.^67^
	*Lactobacillus rhamnosus* GG (LGG)^a^	anti-PD-1	MC38 tumor-bearing mice	–	↓ Tumor growth (tumor size) ↑ survival rate ↑ CD8^+^ T cells, DCs ↑ IFN-β, IFN-γ, CXCL9, CXCL10, ↑ anti-PD-1 efficacy	Si et al.^68^
	*Fusobacterium nucleatum*^a^	anti-PD-1	CT26 tumor-bearing mice	human DLD1, Caco-2	↓ tumor growth (tumor weight, tumor volume) ↑ survival rate ↑ CD8^+^ T cells, IFN-γ, PD-L1, NF-κB ↑ anti-PD-1 efficacy	Gao et al.^69^
	gasdermin E (GSDME)^a^	anti-PD-1	MC38 and CT26 tumor-bearing mice	human HT29, HCT116, SW480, SW620 Murine CT26, MC38	↓ tumor growth (tumor weight and volume) ↑ CD8^+^ T cells, IL2, TNFα, IFNβ, Granzyme B, Perforin, ↑ anti-PD-1 efficacy	Luo et al.^70^
	trichosanthin^a^	anti-PD-1	CT26 tumor-bearing mice	–	↑ CD8^+^ T cells, CD4^+^ T helper cells, IFNγ, granzyme B ↓ PD-L1, M2 macrophages, Tregs ↑ anti-PD-1 efficacy	Zhang et al.^71^
	TMPyP4^a^	anti-PD-1	PDX (a CRC patient-derived xenograft) model MC38 tumor-bearing mice	human HCT116, DLD1, HCT8, HCT15, LOVO, SW620, CACO2, LST174T, RKO, NCI-H508 murine CT26, MC38	↓ tumor growth (tumor volume, tumor weight) ↑ patient survival ↑ CD8^+^ T cells, DCs ↑ CCL5, CXCL10, IFNβ ↑ anti-PD-1 efficacy	Li et al.^72^
	CX-5461^a^	anti-PD-1 anti-PD-L1	CT26 tumor-bearing mice	human HT29, DLD1 murine CT26	↓ tumor growth (tumor volume) ↑ survival time ↑ IFN-α, IFN-β, CCL5, and CXCL10, ↑ CD4^+^ and CD8^+^ T cells, Treg cells ↓ MDSCs ↑ anti-PD-1, anti-PD-L1 efficacy	Chung et al.^73^
	*Bifidobacterium*^a^	anti-CD47 immunotherapy	CT26 tumor-bearing mice	–	↓ tumor growth (tumor volume) ↑ IFN-β, IFN-γ ↑ local anti-CD47 immunotherapy efficacy	Shi et al.^74^
	lovastatin (SHP2 agonist)^a^	–	CT26 tumor-bearing mice	human HCT116, HT29, SW620	↓ DNA repair via the dephosphorylation of PARP1	Wei et al.^75^
		–	HCT116 tumor-bearing mice	–	↓ tumor growth (tumor weight and volume) ↑ tumor cell death ↑ type I IFNs gene (*IFNB1*, *IFIT3, ISG15,* and *IFIT1*)	Huang et l.^76^
	CPI-48 (KDM5 [lysine-specific demethylase 5] inhibitor)^a^	–	MC38, CT26 tumor-bearing mice	–	↓ tumor growth (tumor volume)	Zheng et al.^77^
	CS/NPs (pH-sensitive zeolitic imidazolate framework-8)^a^	–	CT26-Luc tumor-bearing mice	human RKO murine CT26	↓ tumor growth (volume) ↑ CD8^+^ and CD4^+^ T cells, DCs ↑ granzyme B, TNF-α, IL-2, IL-6, IFN-γ ↓ PD-L1, Tregs	Zhang et al.^78^
	NPs (an ROS-sensitive polymer (P1) and mPEG2k-DSPE into ROS responsive nanoparticles)^a^	–	CT26 tumor-bearing mice	murine CT26, MC38	↑ tumor cell death ↑ IFN-β, IL-6, IFN-γ ↑ CD8^+^ T cells, DCs	Cao et al.^79^
	Cur/L-OHP@HAP NPs (hydroxyapatite nanoparticles co-loaded with curcumin and L-oxaliplatin)^a^	–	CT26 tumor-bearing mice	–	↓ tumor growth (tumor weight and volume) ↑ CD8^+^ T cells, DCs, IFN-β, IL-6, TNF-α ↓ Tregs	Xiao et al.^80^
	Ara-C^a^	–	–	human HCT116, HT29	↓ tumor growth ↑ IFN-γ^+^ CD8 T cell, NK cell function (MICA/B and ULBP2/5/6)	Lewicky et al.^81^
	ESCu@HM-induced cuproptosis^a^	–	CT26 tumor-bearing mice	human THLE-2 murine CT26, CT26-Luc	↓ tumor growth (tumor volume, tumor weight) ↑ survival time ↑ CD4^+^ and CD8^+^ T cells, DCs, M1 macrophages ↑ IFN-β, IL-6, and TNF-α	Li et al.^82^
	CS-HAP@KAE^a^	–	CT26 tumor-bearing mice	CT26 cells and human FHC (epithelial cell lines of fetal)	↑ tumor cell death ↑ CD4^+^ and CD8^+^ T cells, M1 macrophages ↑ IL-1β, IL-18, IFN-β, NLRP3/caspase-1/GSDMD ↓ M2 macrophages, Tregs	Chen et al.^83^
	talazoparib (PARPi) and palbociclib (CDK4/6i)^a^	–	HCT116, CT26 tumor-bearing mice	human HCT116, LoVo Murine CT26-LUC, MC38	↓ tumor growth (tumor volume) ↑ IFN-β, CCL5, CXCL10, IL-1β, IL-6, and TNF-α	Wang et al.^84^
	cGAMP^b^	–	MC38 tumor-bearing mice	–	↓ liver CRC metastasis ↑ IL-18 and IL-1β by macrophages ↑ anti-tumor activity of NK cell	Sun et al.^85^
		–	AOM/DSS mouse model	–	↓ tumor growth (tumor number and tumor size) ↑ IFN-α, IFN-β, and CXCL10	Hu et al.^86^
		–	CT26 tumor-bearing mice	–	↓ tumor growth (tumor size), ↑ survival rate ↑ CD8^+^ T cells, macrophages ↑ TNF-α, CXCL10, CXCL11, IFN-induced molecules by macrophage	Ohkuri et al.^87^
		–	MC38 tumor-bearing mice	–	↓ tumor growth (tumor volume) ↑ survival rate ↑ CD8^+^ T cells	Chon et al.^88^
		–	Zebrafish xenograft model	–	↓ tumor growth and metastasis ↑ IL-2, TNF-α, IFN-γ ↓ CXCL8, BCL-2, VEGFA	Jiang et al.^89^
	2′3′-cGAMP and DMXAA^b^	–	AOM/DSS mouse model	human HT29	↓ CRC tumorigenesis ↓ tumor growth (tumor number) ↑ tumor cell death	Gong et al.^90^
	DMXAA^b^	–	MC38 tumor-bearing mice	human DLD1 murine MC38, CT26	↓ liver metastasis ↑ the survival rate ↑ M1 macrophages	Liu et al.^91^
	ADU-S100^b^	oxaliplatin	CT26 tumor-bearing mice	–	↓ tumor growth (tumor volume) ↑ survival rate ↓ Treg cells, M2 macrophages ↑ CD8^+^, CD4^+^ T cells, DCs ↑ IL-2, IFN- γ, and TNF-α	Gu et al.^92^
		reovirus	CT26 tumor-bearing mice	murine CT26, MC38, CMT93	↓ tumor growth (tumor volume) ↑ survival time ↑ CD8 cells, M1 macrophages ↑ granzyme B, IFN-β ↓ Tregs	Sugimura et al.^93^
	cGAMP, RR-CDA (MIW815), and ADU-S100^b^	VEGRF2 blockade immune-checkpoint blockade (αPD-1 or αCTLA-4)	CT26 tumor-bearing mice	–	↓ tumor growth (tumor volume) and distant metastasis normalized tumor vasculature and the tumor microenvironment. ↓ lymphovascular invasion ↑ CD8^+^ T cells, macrophage M1 ↑ type I/II IFN genes and vascular stabilizing genes (e.g., *Angpt1*, *Pdgfrb*, and *Col4a*) and adhesion molecules, including Icam, Vcam, and Sell	Yang et al.^94^
	PolySTING^b^	anti-PD-1	MC38 tumor-bearing mice	murine MC38	↓ tumor growth (tumor volume, tumor weight), metastasis ↑ CD8^+^ T cells, DCs, macrophages, and NK cells ↑ IFN-γ, granzyme B, CCL4/5, CXCL9/10 ↑ anti-PD-1 efficacy	Wang et al.^95^
	diAMP-BCM (a STING agonist-loaded CuS/MnO2 bimetallic nanosystem)^b^	anti PD-1	MC38 and CT26 tumor-bearing mice	–	↓ tumor growth (tumor volume) ↑ tumor cell death ↓ T cell exhaustion (TIM3^+^ T cells) ↑ CXCL10, IFN-β, M1 macrophages, CD8^+^ T cells ↑ anti-PD-1 efficacy	Peng et al.^96^
	diABZIs (two symmetry-related amidobenzimidazole [ABZI]-based compounds to create linked ABZIs)^b^	–	CT26 tumor-bearing mice	–	↓ tumor growth (tumor volume) ↑ survival time ↑ IFN-β, IL-6, TNF, CXCL1	Ramanjulu et al.^97^

Gy, gray; Trp53, transformation-related protein 53; TGF-β, transforming growth factor beta; Alk5, activin receptor-like kinase 5; Tgfbr1, transforming growth factor beta receptor 1; AOM, azoxymethane; DSS, dextran sulfate sodium; COX-2, cyclooxygenase-2; OVA, ovalbumin; TIM3, T-cell immunoglobulin and mucin domain-containing protein 3; TAM, tumor-associated macrophage; ATM, ataxia telangiectasia mutated; PREX2, phosphatidylinositol-3,4,5-triphosphate-dependent Rac exchange factor 2; ATR, ataxia telangiectasia mutated and Rad3-related; CTL, cytotoxic T lymphocyte; MHC-1, major histocompatibility complex class 1; FLT3, Fms-like tyrosine kinase 3; TREX-1, three-prime repair exonuclease 1; PARP1, poly(ADP-ribose) polymerase 1; MICA/B, MHC class 1 chain-related protein A and B; ULBP, UL16-binding proteins; PSGL-1, P-selectin glycoprotein ligand-1; JNK, c-Jun N-terminal kinase; STAT1, signal transducer and activator of transcription 1; CRC, colorectal cancer; cGAMP, cyclic GMP-AMP; TRAF6, TNF-receptor-associated factor 6; IRF7, interferon regulatory factor 7; NF-κB, nuclear factor κB; NK, natural killer; Treg, regulatory T cell; MDSC, myeloid-derived suppressor cell; DC, dendritic cell; IFN, interferon; TNF, tumor necrosis factor; BCL-2, B cell lymphoma 2; VEGRF2, vascular endothelial growth factor receptor 2; CTLA-4, cytotoxic T-lymphocyte-associated protein 4; VEGFA, vascular endothelial growth factor A; CCL, C-C motif chemokine ligand; CXCL, C-X-C motif chemokine ligand; NLRP3, NOD-like receptor family pyrin domain containing 3; GSDMD, gasdermin D; SHP2, Src homology-2 domain-containing protein tyrosine phosphatase-2; PD-1, programmed cell death protein 1; PD-L1, programmed cell death ligand 1.

a Indirect cGAS/STING enhancer.

b Direct STING agonist.

### cGAS/STING signaling pathway in RT

#### Role of cGAS/STING in the RT response

The cGAS/STING pathway acts as a critical detector of RT-induced cellular stress.^98^ When DNA damage from RT leads to the accumulation of DNA in the cytosol, this pathway is activated,^65^ ultimately resulting in increased production of inflammatory cytokines such as IFNB1 in the CRC environment.^99^^,^^100^ In a healthy state, DNA is restricted to the mitochondria and nucleus, with any DNA found in the cytosol or endo-lysosomal compartments typically degraded by nucleases. In contrast, DNA accumulates in the cytoplasm during infections or cellular damage, where it can be detected by cGAS. When cGAS interacts with DNA, this event triggers a structural rearrangement that switches on its enzymatic function. Activated cGAS then catalyzes the synthesis of cyclic GMP-AMP (cGAMP) from the substrates guanosine triphosphate (GTP) and adenosine triphosphate (ATP), resulting in the production of this secondary messenger. The resulting cGAMP molecule then attaches to STING, a signaling adaptor embedded in the endoplasmic reticulum (ER) membrane.^101^ Following activation, STING relocates to the Golgi apparatus, where it triggers a series of downstream signaling events.^102^^,^^103^ Typically, STING, when activated by cGAS, stimulates the expression of IRF3 (interferon regulatory factor 3) and TBK1 (TANK-binding kinase 1), ultimately resulting in IFNB1 production.^104^ IFN secretion enhances the cytolytic potency of CD8^+^ T cells while simultaneously pushing DC maturation. Alternatively, STING can recruit IκB kinase to initiate the NF-κB signaling cascade, which drives inflammatory responses.^105^ Exposure to ionizing radiation often leads to DSBs in the DNA of cancer cells. Errors during the repair process can cause segments of nuclear double-stranded DNA (dsDNA) to collect in the cytoplasm, sometimes within micronuclei. cGAS recognizes these cytosolic DNA fragments, subsequently triggering the activation of the STING signaling pathway.^106^ Overall, cGAS/STING signaling detects aberrant DNA and converts this signal into immune activation directed at tumor targets.

RT induces DNA damage and triggers anti-cancer immune activity through the stimulation of cGAS/STING signaling. dsDNA fragments often associate with phosphorylated histone H2AX (γH2AX), a key marker that facilitates the activation of cGAS. Following its activation, cGAS synthesizes cGAMP, which acts as a secondary messenger. cGAMP then associates with the STING protein residing on the endoplasmic reticulum membrane.^107^^,^^108^ This interaction triggers a cascade of gene expression that increases levels of pro-inflammatory cytokines and IFN-stimulated genes. As a result, immune cells such as T cells and natural killer cells are mobilized, strengthening the body’s collective response against tumor cells.^101^^,^^109^^,^^110^ Through effectively recruiting tumor-targeting CD8^+^ T cells and other immune cells,^88^^,^^111^ this process amplifies the immune response, ultimately aiding in tumor elimination.^100^^,^^112^^,^^113^^,^^114^ In the TME, dsDNA released by tumor cells via exocytosis triggers the cGAS/STING activation, facilitating maturation, polarization, and differentiation of macrophages and DCs.^115^^,^^116^ cGAS/STING signaling enhances T cell function and promotes the cell death of malignant B cells in the TME by regulating their differentiation and apoptosis.^117^^,^^118^^,^^119^ Elevated STING expression correlates with improved prognosis in CRC patients, reflecting its role in fostering a favorable immune response and improving treatment outcomes.^88^^,^^120^ Collectively, cGAS/STING signaling aids tumor suppression by amplifying the immune response. Activation of this pathway enhances the diversity of immune cells within the TME and encourages TAMs to adopt a pro-inflammatory phenotype. Through these processes, the cGAS/STING axis serves as a crucial link connecting the detection of DNA damage to the coordination of both innate and adaptive immune responses (Figure 1).

On the other hand, the fact that cGAS/STING signaling in CRC can exert divergent, context-dependent effects poses a challenge for rational drug design. Even so, converging evidence from experimental models and initial clinical investigations indicates that deliberate activation of the canonical cGAS/STING/IRF3/IFNB1 axis can potentiate standard chemotherapy and immune checkpoint blockade, and this enhancement is frequently linked to better clinical responses and more favorable prognostic profiles.^120^^,^^121^ By contrast, diminished therapeutic efficacy linked to the cGAS/STING axis appears in contexts where noncanonical signaling predominates, for example via the AMPK (adenosine-monophosphate-activated protein kinase)/mTOR (mammalian target of rapamycin) cascade.^122^ Such noncanonical signaling downstream of cGAS/STING enables cancer cells to endure and continue proliferating, thereby sustaining malignant progression. In parallel, long-lasting cGAS/STING activation within immune cells can fuel a persistent, Th1-skewed inflammatory milieu that fails to resolve, a process increasingly linked to the evolution of IBD into CRC.^113^^,^^123^^,^^124^^,^^125^ Conversely, deficiency of cGAS^86^ or STING^90^ within CRC cells markedly increases susceptibility to IBD-driven tumorigenesis *in vivo*. Notably, in the AOM/DSS model that mimics chronic-colitis-driven CRC, levels of DEAD-box helicase 41 (DDX41), a dsDNA sensor that relays signals through STING to trigger IFN-1 production,^126^ were found to be diminished.^127^ Correspondingly, C5aR1 suppresses STING-driven IFN-1 production via DDX41 during acute inflammatory responses to infection.^128^ In addition, inhibition of C5aR1 was shown to enhance CRC treatment in AOM/DSS mouse models.^35^ These observations raise an intriguing question: within a chronically inflamed or immunosuppressive TME, does activating the canonical immune-response pathway in CRC cells promote anti-tumor clearance or instead support immune escape? Moreover, the detailed molecular basis underlying the paradoxical, tumor-suppressive versus tumor-promoting roles of the cGAS/STING cascade in CRC remains poorly defined. Thus, investigating the interplay between cGAS/STING signaling and C5aR1 in CRC progression and therapy may help distinguish circumstances in which the classic IRF3/IFN-1 pathway promotes immune-mediated tumor suppression, versus scenarios where non-canonical signaling leads to tumor cell elimination. Such knowledge would be valuable for refining and improving therapeutic strategies for CRC.

#### Enhancement of cGAS/STING to improve CRC treatment outcomes

cGAS/STING signaling has emerged as a contributor to chemoresistance in CRC following treatment with conventional regimens such as RT. RT causes DNA damage by generating highly reactive free radicals that result in DSBs. DNA lesions initiate a series of DNA damage response (DDR) pathways, frequently through the involvement of PI3K and related kinases including ATR (ataxia-telangiectasia and Rad3-related), ATM (ataxia-telangiectasia mutated), and DNA-PKcs (DNA-dependent protein kinase catalytic subunit).^129^ These kinases subsequently trigger the activation of checkpoint kinase 1 (Chk1), predominantly via ATR, and checkpoint kinase 2 (Chk2), primarily via ATM. Activation of these signaling proteins slows cell-cycle progression, providing an opportunity for DNA repair mechanisms to function efficiently.^130^^,^^131^ In particular, ATM-dependent Chk2 activation is commonly triggered by RT -induced DSBs. In parallel, PI3K/AKT signaling modulates cell-cycle dynamics and suppresses apoptosis, which speeds the resolution of radiation-generated DSBs and, as a consequence, enhances the capacity of tumor cells to withstand radiation.^132^ PI3K/AKT overexpression drives tumor progression and correlates with adverse outcomes following RT. Despite the potential of targeting this pathway for cancer treatment, instances of chemoresistance have been observed. Moreover, in hepatocellular carcinoma, cGAS acts as an anti-tumor regulator by facilitating Src homology region 2-containing phosphatase 1 (SHP1)-mediated dephosphorylation of the PI3K p85 regulatory subunit, thereby dampening the PI3K/AKT/mTORC1 pathway.^133^ Thus, the emergence of this drug resistance could stem from disruptions or insufficiency in the cGAS/STING pathway, yet its significance in CRC remains to be thoroughly examined. In terms of EGFR (epidermal growth factor receptor) activity, intrinsic activation of this molecule initiates downstream signaling that enhances tumor proliferation and survival, which in turn reduces the effectiveness of chemotherapy and RT.^134^ Therefore, inhibition of EGFR offers a potential approach to bypass resistance and improve CRC treatment outcomes. However, EGFR-mediated phosphorylation of cGAS/STING at a tyrosine site is essential for directing STING to the endosomes, facilitating its interaction with IRF3. When EGFR-mediated phosphorylation is lacking, STING moves swiftly to autophagosomes, leading to reduced IRF3 activation and IFN-1 production, while simultaneously promoting autophagy in cell and animal models.^135^ This shift in STING localization and signaling under EGFR blockade may contribute to resistance to EGFR-targeted therapy in CRC. The altered positioning and signaling of STING that occur when EGFR is inhibited might play a role in the development of resistance to therapies targeting EGFR in CRC. This finding points to an underappreciated resistance mechanism that deserves deeper exploration.

An intact, well-functioning cGAS/STING axis may be essential for reducing heterogeneity in therapeutic responses among patients with CRC. CRC patients with better survival showed higher STING expression.^120^^,^^121^ Conversely, low STING levels have been linked to weaker RT outcomes and reduced survival rates in CRC.^136^ Ionizing radiation in CRC is capable of activating cGAS/STING, triggering IFN-1 and inflammatory cytokines that foster abundant intratumoral cytotoxic CD8^+^ T cell infiltration. When tumor cells are exposed to radiation, activation of the cGAS/STING pathway leads to the synthesis of cGAMP. This molecule acts as a signaling messenger, capable of transmitting activation cues to neighboring immune cells such as DCs, CD8^+^/CD4^+^ T cells, and macrophages.^87^^,^^109^^,^^137^^,^^138^ As these signaling molecules propagate across the tumor microenvironment, they intensify STING pathway activation within nearby immune cells. This escalation supports the arrival, persistence, and activation of various immune cell types in the tumor. The resulting environment is marked by heightened IFN-1 responses and increased expression of IFN-stimulated genes, creating a more inflammatory and immune-responsive tumor landscape.^87^^,^^137^^,^^138^ However, one study found reduced CRC cell proliferation following STING knockdown, suggesting that higher STING levels may promote CRC proliferation. Additionally, elevated STING expression is observed in CRC cases exhibiting lymph node metastasis compared to those without.^122^ In this work, STING was found to drive CRC progression via an unconventional signaling route, marked by suppression of AMPK (AMP-activated protein kinase) and concomitant activation of the mTOR cascade in cultured cells. Nevertheless, the examined samples, selected based on lymph node metastasis and different stages of cancer progression, may not accurately reflect the association between STING and prognosis following CRC treatment.^122^ Additionally, the authors used HCT116 and SW480 cell lines characterized by cGAS deficiency, which may not adequately represent STING functions in this context.^98^ In contrast, a previous study found that CRC patients with lower STING expression after chemotherapy and RT had lower survival rates.^136^ The authors have shown that the HCT116 and SW480 cell lines, which have lower cGAS expression, exhibit reduced apoptosis after ionizing radiation compared to the HT29 cell line, which is cGAS-proficient. Together, these observations suggest that intact cooperation between cGAS and STING is indispensable for the STING axis to reshape the TME to achieve beneficial therapeutic outcomes in CRC.

The cGAS/STING pathway plays a central role in orchestrating the repair of DNA DSBs generated by radiation, a frequent form of genomic injury in CRC. The DDR to these breaks is critical for determining patient prognosis, as it significantly influences treatment outcomes.^139^ In up to 20% of CRC cases, somatic mutations affect genes responsible for homologous recombination (HR) repair of DSBs.^140^ The cGAS/STING axis closely interfaces with DSB repair, influencing both non-homologous end joining (NHEJ) and HR, the principal pathways that determine how CRC cells cope with RT-induced DNA breaks.^141^^,^^142^ In several tumor types, including CRC, cGAS/STING signaling has been implicated as a brake on efficient repair of damaged DNA. cGAS may limit the efficiency of homologous recombination repair through two complementary pathways. In the nucleus, cGAS localizes to DNA break sites and associates with PAR polymerases (PARPs) and DNA damage markers such as γH2AX, disrupting the normal assembly of PARP-dependent repair complexes and thereby reducing HR-mediated repair.^143^^,^^144^ Separately, cGAS/STING-generated cGAMP can deplete intracellular nicotinamide adenine dinucleotide (NAD)^+^, thereby limiting the NAD^+^ pool required for poly(ADP-ribose) chain formation. This shortage of substrate curtails poly(ADP)-ribosylation and indirectly suppresses PARP enzymatic function, ultimately further weakening HR-mediated DNA repair.^130^ cGAS can also inhibit Rad51-mediated strand invasion by binding to DNA and undergoing self-oligomerization, which compacts the bound template dsDNA into a higher-order, ladder-like structure.^145^ This conformation makes DNA less accessible for strand invasion by Rad51.^145^ To inhibit NHEJ, cGAS binds to exposed telomeres and attracts cyclin-dependent kinase 1 (CDK1), which suppresses the recruitment of the NHEJ factors RNF8 (RING finger protein 8) and 53BP1 (p53-binding protein 1), thereby preventing chromosome end-to-end fusions.^146^

In contrast, STING can promote DNA repair both dependent and independent of cGAS. STING can influence DNA DSB repair in breast cancer cells through a route that does not rely on cGAS or its classic pro-inflammatory cascade.^147^ A distinct pool of STING is positioned within the nucleus, where it engages components of the DNA-PK complex, stabilizing these factors and supporting the assembly of 53BP1 repair foci. Through this nuclear interaction, STING enhances NHEJ in a mechanism distinct from cGAS signaling.^147^ Moreover, exposure to ionizing radiation engages the cGAS/STING cascade alongside ATM signaling, together driving TBK1- and ATM-dependent phosphorylation of phosphoribosyl pyrophosphate synthetase 1 and 2 (PRPS1/2).^148^ This modification increases PRPS1/2 activity, expanding the pool of deoxyribonucleotide triphosphates (dNTPs) available in the cell. The enriched dNTP supply provides the essential substrates for DNA polymerases, thereby facilitating DNA synthesis during repair and supporting multiple repair processes, including both NHEJ and HR, in irradiated cells.^148^ These multifaceted STING functions add an additional layer of complexity to how it influences CRC development.

The tumor-suppressive influence of cGAS/STING signaling in CRC can be strengthened by its ability to drive programmed cell death. Upon initiation of this pathway, TBK1 is recruited and activated, resulting in the phosphorylation of IRF3. Once phosphorylated, IRF3 is capable of triggering apoptosis via pathways independent of both pro-inflammatory cytokine signaling and p53-mediated mechanisms.^149^ During prolonged mitotic arrest, only a small pool of phosphorylated IRF3 builds up, insufficient to drive IFN expression. Rather than functioning in the nucleus, the altered IRF3 translocates to the mitochondria, where it interferes with the anti-apoptotic protein Bcl-xL (B cell lymphoma-extra large).^149^^,^^150^ By interfering with Bcl-xL’s role at the mitochondrial outer membrane, IRF3 increases the membrane’s permeability. This enhanced permeability enables both cytochrome *c* and SMAC (second mitochondria-derived activator of caspases) to be released from the mitochondria into the cytosol. Once released, these mitochondrial factors cooperate to activate the effector caspases 3, 7, and 9, thereby engaging the intrinsic apoptotic cascade.^149^^,^^150^^,^^151^

In summary, modulating cGAS/STING signaling could enhance RT efficacy in CRC. Moreover, coordinated expression of cGAS and STING might be required for effective and stable cGAS/STING function by hindering the tumor promoter role of the non-canonical STING pathway. How activation of the cGAS/STING pathway influences therapeutic success in CRC remains unclear at this stage. Further comprehensive research is necessary to determine optimal approaches for leveraging this signaling axis in clinical settings.

## Other signaling pathways in RT

A range of signaling pathways has been investigated for their roles in modulating RT response in CRC. Among the various signaling networks involved, the PI3K/AKT/mTOR axis emerges as a central driver of radioresistance. By coordinating key cellular programs, including DNA damage surveillance and repair, cell-cycle progression, uncontrolled proliferation, regulation of programmed cell death, and promotion of invasive behavior, this pathway strongly favors the persistence and survival of irradiated tumor cells. Targeting PI3K/AKT/mTOR in combination with RT can sensitize cancer cells by curbing their survival, promoting apoptosis, arresting the cell cycle, limiting proliferative capacity, and attenuating migratory and metastatic behavior.^152^^,^^153^ Research has shown that ionizing radiation can activate the PI3K/AKT pathway, driving SOX2 (SRY-box transcription factor 2) expression and expanding the CD44^+^ cancer stem-like population in CRC cells, thereby contributing to radioresistance. Blocking PI3K with LY294002 or reducing AKT expression suppresses the radiation-induced rise in SOX2 and CD44, limiting stem-like traits such as sphere formation and invasion.^154^ In addition, the dual PI3K/mTOR inhibitor BEZ235 increases CRC radiosensitivity by elevating DNA DSBs (γ-H2AX) and impairing key DNA repair proteins, including ATM and DNA-PKcs, ultimately restricting tumor cell growth *in vitro*.^153^

Another pathway influencing RT response in CRC is the mitogen-activated protein kinase (MAPK)/extracellular signal-regulated kinase (ERK) signaling. Elevated B7-H3, which can enhance NF-κB, STAT3, or MAPK activity, promotes radioresistance, whereas its depletion increases radiosensitivity.^155^^,^^156^^,^^157^^,^^158^^,^^159^^,^^160^ Mechanistically, they demonstrated that B7-H3 upregulates KIF15 (kinesin family member 15), which then activates ERK1/2 after radiation.^161^ FRA-1 (Fos-related antigen 1), a downstream target of MAPK/ERK in CRC,^162^ appears to promote radioresistance by supporting DNA repair and cell survival after irradiation.^163^ SW480 cells display higher intrinsic resistance to irradiation than SW620, which are comparatively more radiation-sensitive. When FRA-1 expression is knocked down in SW480 cells, their susceptibility to radiation increases markedly, whereas enforcing FRA-1 expression in SW620 cells shifts them toward a more radioresistant phenotype.^163^

Additionally, TGF-β levels were elevated in tissues following RT, correlating with increased metastatic potential.^164^ Experimental evidence indicates that exposure to ionizing radiation activates the TGF-β signaling cascade in CRC cells, leading to a marked upregulation of podocalyxin (PODXL) expression. Elevated PODXL levels enhance migratory capacity and invasive behavior, thereby contributing to radiation tolerance.^164^ Pharmacological inhibition of TGF-β signaling using galunisertib, as well as genetic suppression of PODXL, significantly impaired CRC cell movement and reduced cellular survival following irradiation. These findings suggest that disruption of the TGF-β/PODXL axis may potentiate RT-induced cytotoxicity while simultaneously limiting aggressive tumor phenotypes.^164^ Clinically, combining galunisertib, a TGF-β receptor 1 inhibitor, with standard neoadjuvant chemoradiotherapy yielded a 32% complete response rate in a single-arm phase 2 trial in locally advanced rectal cancer, supporting further evaluation in randomized studies.^165^

Notch1 signaling also contributes to radioresistance in CRC. Targeting Notch1 in CRC cells increases their vulnerability to radiation, in part by relieving Notch1-mediated inhibition on the cyclin-dependent kinase inhibitor p27. This elevation of p27 hampers cell-cycle advancement and reduces cellular growth rates.^166^^,^^167^ Exposure of CRC cells to honokiol, a γ-secretase inhibitor, together with irradiation, suppresses Notch pathway activity and lowers their ability to form colonies.^168^ At the same time, persistent Wnt/β-catenin signaling underlies radioresistant CRC phenotypes. Radioresistant CRC cell lines display heightened amounts of nuclear β-catenin and increased TCF (T cell factor)/LEF (lymphoid enhancer factor)-mediated gene expression. These changes coincide with features resembling epithelial-mesenchymal transition, greater expression of stem-cell-associated markers such as ALDH1 (aldehyde dehydrogenase 1) and CD44, and a rise in invasive behavior.^41^^,^^169^^,^^170^^,^^171^ Mechanistically, Wnt/β-catenin promotes radioresistance by upregulating LIG4 (DNA ligase 4), which reduces γH2AX foci and enhances DSB repair through the NHEJ pathway.^172^

Although several signaling pathways have been investigated for their roles in RT, direct evidence linking these pathways to CRC treatment response remains limited. C5aR1 is gaining prominence as a promising lever to improve RT outcomes; however, the precise mechanisms by which it shapes tumor radiosensitivity remain only partially defined. Among pathways implicated in RT effects, cGAS/STING is a critical radiation sensor and has a recognized connection with C5aR1 in innate immune responses in an infectious context. Interestingly, cGAS/STING and C5aR1 appear to have opposing roles in CRC progression and radiosensitivity. Accordingly, this review examines how cGAS/STING signaling might underlie the radiosensitizing activity of C5aR1 inhibition, with the goal of identifying mechanistic insights that could be leveraged to improve RT efficacy in CRC.

## Potential cGAS/STING-C5aR1 interaction in enhancing CRC radiosensitivity

### Opposing roles of C5aR1 and cGAS/STING in immunotherapy effectiveness

The upregulation of C5aR1 and deficiency of cGAS/STING adversely impact immune therapy outcomes, indicating that their interaction may contribute to T cell dysfunction and immune modulation.

#### C5aR1 impedes immunotherapy effectiveness

C5aR1 activation contributes to an immunosuppressive milieu and correlates with poor responses to immune checkpoint (ICP) therapy in several malignancies, including CRC.^47^^,^^173^^,^^174^^,^^175^^,^^176^^,^^177^^,^^178^^,^^179^ When C5aR1 is activated, it favors the emergence of an immunosuppressive tumor milieu by downregulating CXCL9 (C-X-C motif chemokine ligand 9) and inducing macrophage secretion of immunomodulatory mediators such as TGF-β, IL-10, IL-6, and IL-4. This altered cytokine balance favors M2-like macrophage polarization and impairs macrophage-mediated trafficking and effector activity of cytotoxic T cells, collectively weakening immune surveillance and enabling tumor expansion.^174^^,^^175^^,^^179^ As expected for an immunosuppressive pathway, intensified C5aR1 engagement aligns with compromised T cell immunity, evidenced by the enrichment of exhaustion-associated receptors TIM3 (T cell immunoglobulin and mucin-domain containing 3), LAG3 (lymphocyte-activation gene 3), PD-1 (programmed cell death protein 1), and CTLA4 (cytotoxic T-lymphocyte antigen 4), on CD8^+^ T cells within the tumor microenvironment.^174^^,^^175^ In contrast, disrupting C5aR1 function, either through genetic deletion or chemical inhibition, restores robust T-cell-driven immunity against tumors. This intervention results in a greater number and proliferation of CD4^+^ and CD8^+^ T cells within tumors, reduced expression of immune checkpoint molecules, and heightened cytotoxic activity by these effector cells.^174^^,^^175^ By enhancing the cytotoxic potential of CD8^+^ T cells and encouraging the presence of M1-type, tumor-killing macrophages, inhibition of C5aR1 alters the tumor immune landscape. In this reprogrammed environment, therapies that block PD-1 demonstrate improved anti-cancer efficacy. Additionally, it suppressed key immunosuppressive components of the TME, including Treg cells, MDSCs, exhausted CD8^+^ T cells, and tolerogenic DCs.^47^^,^^174^^,^^175^^,^^179^^,^^180^^,^^181^ C5aR1 blockade led to a broad immunomodulatory shift. It suppressed immunosuppressive cytokines, including IL-10, CXCL1, PGE2 (prostaglandin E2), and TGF-β, and downregulated co-inhibitory receptors (e.g., LAG3 and PD-1). Conversely, it upregulated immune effectors (e.g., granzyme B, IFN-γ, and perforin-1) and proinflammatory cytokines (IL-18, IL-12, and IL-2), suggesting activation of anti-tumor immune pathways.^35^^,^^37^^,^^46^^,^^182^ In line with these observations, treating CRC-bearing mice with both a C5aR1 inhibitor and anti–PD-1/PD-L1 antibodies led to a notable increase in CD8^+^ T cell infiltration within tumors and substantially elevated IFN-γ release from both CD4^+^ and CD8^+^ T cells.^182^ Thus, C5aR1 deletion or blockade could reprogram macrophages and reinvigorate anti-tumor T cells, overcoming resistance to ICP therapy and significantly slowing CRC progression.

C5aR1 has been implicated in sustaining PD-1/PD-L1 expression and an immunosuppressive milieu in refractory CRC, yet the detailed mechanisms remain largely unresolved. Notably, ovarian tumors with high expression of both C5aR1 and PD-L1 display increased activation of the PI3K and Janus kinase/signal transducers and activators of transcription (JAK/STAT) pathways.^175^ These signaling cascades are also recognized as upstream regulators of PD-1 and PD-L1 expression in CRC.^183^^,^^184^ In addition, when C5aR1 expression is reduced in tumors, there is a marked increase in IFN-γ signaling,^21^^,^^35^^,^^37^^,^^47^ an important stimulus for activating the cGAS/STING pathway.^185^ C5a/C5aR1-driven PD-L1 expression on human monocytes is mediated in part by p38 MAPK/NF-κB, and additionally by JNK (Jun N-terminal kinase) and ERK1/2 signaling.^186^ Furthermore, administering PD-1 or PD-L1 antibodies to colorectal tumors increases the generation of C5a in the TME, which in turn heightens the immunosuppressive actions of MDSCs.^182^ As a result, T cell expansion and cytolytic capacity are curtailed, weakening the responsiveness of CRC mouse models to checkpoint inhibition.^182^ The C5a/C5aR1 signaling axis reportedly promotes CRC development by enhancing MDSC influx into the TME and polarizing TAMs toward an immune-dampening, tumor-promoting phenotype, in part through monocyte chemoattractant protein-1 (MCP-1)/C-C motif chemokine ligand 2 (CCL2)-mediated engagement of PI3K/AKT pathways in macrophages.^34^^,^^35^^,^^36^^,^^37^^,^^187^ When the PI3K/AKT/mTOR cascade remains chronically active, colorectal tumors tend to respond less favorably to RT, in part due to an enhanced DDR and repair capacity. Conversely, genetic knockdown or pharmacologic inhibition of key pathway components reproducibly sensitizes CRC cell lines and organoids to radiation.^152^^,^^188^^,^^189^^,^^190^^,^^191^ Functioning upstream of PI3K/AKT/mTOR,^192^ PRDM15 drives radioresistance in CRC cells through physical interaction with the DNA-PKcs–Ku70/Ku80 complex, which in turn facilitates DSB repair via NHEJ pathway^8^ (Figure 1). In addition, pharmacologic cGAMP, acting as an STING pathway modulator, resensitizes oxaliplatin-resistant CRC cells and augments tumor cell killing, at least partly by attenuating PI3K/AKT phosphorylation while boosting p53 expression.^193^ Additionally, blocking the C5a/C5aR1 signaling axis alongside PD-1/PD-L1 inhibitors significantly improves therapeutic responses in CRC.^182^^,^^194^ Therefore, clarifying the immunosuppressive mechanisms orchestrated by C5aR1, with particular attention to its modulation of cGAS/STING, may uncover actionable strategies to circumvent immune resistance in CRC.

#### cGAS/STING promotes immunotherapy effectiveness

Therapeutic cGAS/STING activation synergizes with immune checkpoint inhibition in CRC by promoting anti-tumor immunity.^68^^,^^71^^,^^195^^,^^196^^,^^197^ Agents such as methotrexate (MTX), 5-fluorouracil (5-FU), oxaliplatin, topoisomerase I inhibitors, and riluzole inflict DNA damage that externalizes dsDNA to the TME, where it activates cGAS/STING and reinforces immune-mediated tumor control.^75^^,^^78^^,^^79^^,^^198^^,^^199^^,^^200^^,^^201^ Mitoxantrone-induced dsDNA lesions in CRC cells generate cytosolic DNA that stimulates cGAS/STING signaling, upregulating IFNB1 and inflammatory cytokine production. In parallel, thymus pentapeptide amplifies DC and T cell responses, and zinc-activated AMPK signaling hastens PD-L1 degradation and limits immunosuppressive Treg cells, thereby consolidating anti-tumor immunity.^78^ SN38, a topoisomerase I inhibitor, boosts anti-tumor immunity in CRC by inflicting DNA damage that drives cytosolic DNA buildup, thereby engaging tumor-cell cGAS/STING signaling, elevating IFNB1 and pro-inflammatory cytokines, and ultimately strengthening responses to immune checkpoint blockade.^62^^,^^200^^,^^202^ In CRC, SHP099 intensifies STING/TBK1/IRF3-mediated IFN-1 responses across malignant cells and key immune infiltrates, most prominently macrophages and T cells, thereby slowing tumor progression.^203^ By inducing cytosolic dsDNA accumulation and γH2AX-associated DNA damage, agents such as riluzole, 5-FU, and oxaliplatin effectively trigger cGAS/STING pathway activation. By recruiting and activating NK cells, cytotoxic T cells, and DCs, this response suppresses tumor proliferation in immunosuppressive CRC.^75^^,^^196^^,^^199^^,^^204^ Oxaliplatin with 5-FU triggers cGAS/STING-dependent immunogenic cell death, promotes M1-like, MHC-1-high macrophages, and reduces immunosuppressive cell subsets in the TME.^205^ Dual blockade by talazoparib and palbociclib drives a senescence-oriented cellular state that engages cGAS/STING signaling, promoting dense infiltration of CD8^+^ T cells and NK cells and concurrently reducing macrophage and granulocytic MDSC abundance in the TME.^84^ Furthermore, cGAS/STING activation and strong IFN-1 response occur in CRC cells when cytosolic mitochondrial DNA accumulates after serine deprivation compromises mitochondrial integrity.^206^ This metabolic reprogramming of cGAS/STING axis amplifies IFN-1 signaling, orchestrates DC and CD8^+^ T cell trafficking into tumors, and thereby restrains CRC growth.^206^ Similarly, other STING agonists are known to initiate apoptosis in CRC cells, promote T cell recruitment, drive macrophage polarization, and enhance DC maturation.^96^^,^^207^ DiAMP-BCM nanoparticles selectively stimulate STING/IRF7/CXCL10-mediated inflammatory signaling in macrophages, driving T and NK cell recruitment and attenuating pro-tumor cytokine expression.^96^ Therapies that stimulate cGAS/STING signaling in CRC prompt a robust immune activation. This is evidenced by the heightened presence of inflammatory mediators, such as IL-6, IL-2, CXCL10, CCL5, IFN-γ/β, and TNF-α, alongside an increase in the cytotoxic protein granzyme B within the tumor microenvironment.^78^^,^^84^^,^^196^^,^^199^^,^^200^^,^^204^^,^^206^ These approaches enhanced immune checkpoint blockade efficacy and suppressed tumor growth when combined, with the response dependent on cGAS/STING pathway activation.^84^^,^^196^^,^^200^^,^^205^^,^^206^

RT generates cytosolic dsDNA and micronuclei, stimulating cGAS/STING signaling. IFN-1 and inflammatory cytokine release ignite anti-tumor immunity by mobilizing DCs and cytotoxic T cells,^66^^,^^100^^,^^208^ making RT-induced DNA damage a strong persuasive basis for pairing radiation with immunotherapy. In CRC models, RT combined with TLC388^62^ or irinotecan (IRIN)^66^ silicasomes intensifies DNA lesions, augments cGAS/STING activation, and recruits activated DCs, CD8^+^ effector/memory T cells, and NK cells, ultimately strengthening the efficacy of anti-PD-1/PD-L1 immunotherapy. A regimen combining RT, anti-SIRPα (signal regulatory protein α), and anti-PD-1 harnesses cGAS/STING-driven DC priming to expand tumor-reactive CD8^+^ T cells, enabling complete regression of poorly immunogenic colorectal tumors *in vivo*.^209^ Combining RT with the ATR inhibitor berzosertib results in the accumulation of cytosolic dsDNA, which triggers the cGAS/STING/TBK1/IRF3 signaling cascade. This not only enhances the effectiveness of anti-PD-L1 therapy but also increases the recruitment of CD8^+^ T cells, in part by alleviating SHP1-mediated suppression of STING/TRAF6 (TNF-receptor-associated factor 6)/NF-κB/p65 activity.^65^ Therefore, integrating RT with therapies that induce the cGAS/STING pathway activation can reinstate cancer immunogenicity, facilitating the radiation-driven immunogenic eradication of CRC. Conversely, RT simultaneously fosters the activation of immunosuppressive elements, including MDSCs, TAMs, and Tregs, further complicating the CRC niche.^210^ One possible contributing factor to this phenomenon is the upregulation of C5aR1 activity induced by RT in these tumor-related immune cells.^21^^,^^174^^,^^179^^,^^211^ Importantly, C5aR1 inhibitors have demonstrated potential to enhance the effectiveness of RT in immunosuppressive CRC.^21^ Consequently, an intriguing question emerges as to whether the effects mediated by C5aR1 antagonists are contingent upon cGAS/STING signaling within both CRC cells and the associated immune infiltrates.

cGAS/STING-driven PD-1/PD-L1 upregulation has revealed its capacity to potentiate CRC elimination via ICP therapy. Notably, in PD-L1-negative tumors, CRC patients exhibiting STING-positive tumors experienced a markedly increased 5-year survival rate than those with STING-negative tumors.^212^ Effective anti-tumor responses to PD-L1 blockade rely on an intact cGAS/STING signaling pathway.^111^^,^^213^ Exposure to DNA-damaging regimens, including oxaliplatin, 5-FU, and CX-5461, initiates cGAS/STING/IFN-1 signaling, promoting immune-mediated tumor control while concurrently stimulating PD-L1 upregulation.^73^^,^^199^ Elevated PD-L1 expression on CRC cells may signal an underlying hyperactivated cGAS-STING signaling activity.^69^^,^^199^ STING-driven upregulation of PD-L1 exhausts CD8^+^ T cells, contributing to immune evasion, yet it also presents a targetable axis for improving immunotherapy efficacy.^69^^,^^199^^,^^214^ For instance, the incorporation of anti-PD-1 therapy into a 5-FU/oxaliplatin regimen significantly improved therapeutic outcomes in CRC models, achieving greater tumor reduction than chemotherapy alone.^199^ In CRC models, *Fusobacterium nucleatum* (*F. nucleatum*) enhances STING-orchestrated PD-L1 induction while broadening the pool of IFN-γ-secreting intratumoral CD8^+^ T cells, thereby making PD-L1 blockade more effective against these tumors.^69^ Collectively, these data argue for jointly deploying PD-1/PD-L1 checkpoint blockade and pharmacologic cGAS/STING activation to reinforce anti-tumor immunity and superior CRC management. In summary, current findings advocate for a therapeutic strategy that simultaneously targets the PD-1/PD-L1 pathway and the cGAS/STING axis, with the goal of amplifying anti-tumor immune responses and improving CRC management.

In CRC lesions, C5aR1 blockade may converge on the cGAS/STING axis to modulate immune checkpoint programs, prominently PD-1 and PD-L1, consistent with the capacity of cGAS/STING signaling to boost these checkpoints through diverse downstream circuits. In CRC, its classical cGAS/STING/IRF3 and NF-κB cascades predominantly generate type I IFNs, especially IFN-β, thereby promoting anti-tumor immune activation and fostering IFN-γ secretion by infiltrating immune cells. Additionally, these IFNs, along with their corresponding signaling cascades, such as JAK/STAT1, often driven by IFN-γ within the TME, are essential for orchestrating the upregulation of PD-1 and PD-L1 on malignant and immune cells in CRC^63^^,^^73^^,^^199^^,^^215^^,^^216^ (Figure 1). *F. nucleatum* engages the cGAS/STING axis to activate NF-κB/p65, thereby boosting PD-L1 gene transcription and rendering tumors more susceptible to PD-L1-blockade-mediated anti-tumor immunity.^69^ In cancer progression, elevated C5aR1 expression coincides with increased activation of JAK/STAT signaling.^217^ Furthermore, C5aR1 contributes to CRC metastasis by fostering an immunosuppressive TME, promoting an M2 pattern of macrophages through NF-κB/p65 signaling.^34^ Interestingly, inhibition of C5aR1 selectively induces NF-κB-mediated apoptosis in CRC tumors, while sparing normal tissues.^21^ In the context of NSCLC, RT intensifies C5a/C5aR1 signaling, which activates downstream AKT/IκBα/NF-κB pathways and consequently upregulates pro-inflammatory cytokine genes, particularly IL-6, TNF-α, and IL-12, within irradiated tumors.^218^ This C5a/C5aR1-driven signaling fosters radioresistance, whereas pharmacologic C5aR1 inhibition counteracts this effect and renders tumors more responsive to irradiation.^218^ Collectively, these findings identify C5aR1 as a promising node for therapeutic modulation. Its inhibition may enhance the anti-tumor benefits of cGAS/STING activation while attenuating this pathway’s context-dependent, CRC-associated protumor activities driven by sustained signaling and inflammation.

### Opposing roles of C5aR1 and cGAS/STING in CRC resistance to RT

**Figure 2:**
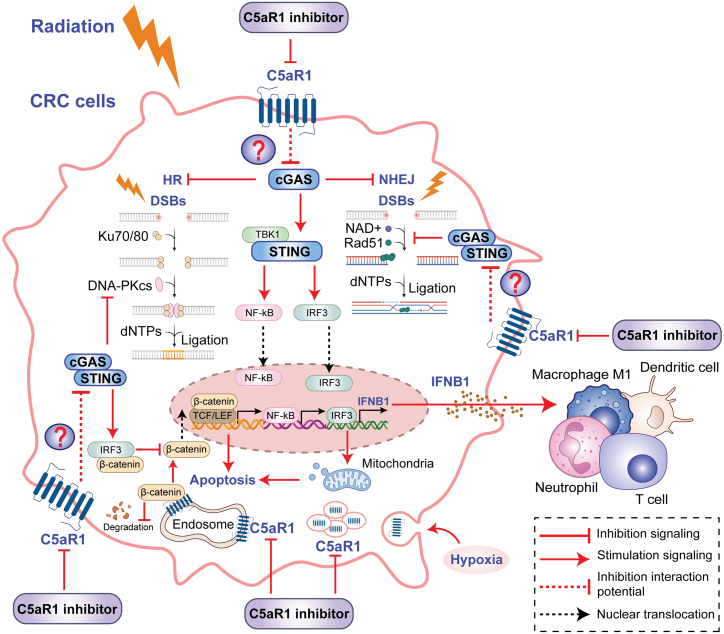
A proposed model for the potential mechanism by which the C5aR1 inhibitor improves radiosensitivity involves the cGAS/STING signaling pathway Enhanced cGAS/STING signaling may contribute to the therapeutic effects of C5aR1 inhibition in CRC treatment. This includes the ability of cGAS/STING to eliminate tumor cells by recruiting immune cells, impairing DNA repair, and inducing intrinsic apoptosis. CRC, colorectal cancer cell; C5aR1, complement component 5a receptor 1; cGAS, cyclic GMP-AMP synthase; STING, stimulator of interferon genes; TBK1, TANK-binding kinase 1; NF-κB, nuclear factor κB; IRF3, interferon regulatory factor 3; DSBs, double-strand breaks; NHEJ, non-homologous end joining; HR, homologous recombination; IFNB1, interferon beta 1; DNA-PKCs, DNA-dependent protein kinase complexs; dNTPs, deoxyribonucleotide triphosphates; NAD+, nicotinamide adenine dinucleotide; Rad51, radiation-sensitive 51; TCF/LEF, T cell factor/lymphoid enhancer factor. Some graphical elements were created using BioRender (Pham, Q. (2026) https://BioRender.com/tf61v6z).

While the impact of C5aR1 antagonists on CRC outcomes is gradually being studied, the mechanisms by which C5aR1 inhibition enhances radiosensitivity in CRC remain unclear. As a central sensor in innate immunity, the cGAS/STING axis coordinates IFN-1-driven defenses against microbial threats. In infectious settings, engagement of C5aR signaling attenuates STING activity, thereby curbing IFN-1 production,^128^ a response that is also pivotal for cGAS/STING-dependent RT efficacy in CRC.^100^^,^^106^^,^^185^ Tumor cells frequently exploit disruption of the cGAS/STING axis and IFN deletion as mechanisms to escape immune surveillance and evade activation.^98^^,^^106^^,^^219^ In this context, harnessing cGAS/STING activation may emerge as a critical effector in potentiating the therapeutic efficacy of C5aR1 inhibition (Figure 2).

In CRC patients with an unfavorable prognosis, defective cGAS/STING signaling or elevated C5aR1 expression has been documented. Increased C5aR1 levels correlate with advanced disease stages and diminished survival prospects,^21^^,^^33^^,^^35^ while C5aR1 deficiency has been shown to impair metastatic potential *in vivo*.^34^ Targeting C5aR1 augments RT efficacy within the immunosuppressive TME, inducing NF-κB-dependent apoptosis exclusively within tumor tissues.^21^ During RT for CRC, engagement of the innate immune cGAS/STING circuitry drives a more immunoreactive tumor bed, characterized by heightened immune-cell recruitment and strengthened tumor-restraining activity.^88^^,^^106^^,^^113^^,^^220^ Reduced expression of cGAS/STING and its signaling components, such as STING, IFNB1, and TBK1, correlates with impaired immune infiltration and increased radioresistance in CRC.^94^^,^^106^^,^^221^^,^^222^ Human CRC cell lines with lower cGAS expression show reduced apoptosis after radiation compared to the cGAS-sufficient cell lines.^98^^,^^223^^,^^224^ Patients with higher cGAS/STING expression have better prognostic outcomes for CRC, whereas low cGAS/STING expression is associated with shorter cancer-specific survival following DNA-damaging therapies.^88^^,^^109^^,^^121^^,^^222^^,^^225^ Moreover, targeting C5aR1 inhibition or cGAS/STING activation has demonstrated significant potential in enhancing CRC treatment, specifically by increasing the radiosensitivity of CRC cells (Table 1).

A higher abundance of TAMs correlates with increased C5aR1 expression and reduced cGAS/STING expression in the TME of CRC. C5a and its C5aR1 receptors, found on immune-suppressive cells such as TAMs in the CRC microenvironment, can stimulate macrophage M2 polarization and promote metastasis.^33^^,^^34^ Moreover, elevated *C5AR1* gene expression, a signature of macrophage-related differentially expressed genes in colon and rectal adenocarcinoma, strongly correlates with increased macrophage M2 infiltration in CRC tumor tissues.^226^ Disruption of the C5a/C5aR1 axis in CRC metastatic foci curtailed M2 macrophage polarization and inflammatory cell recruitment, both implicated in liver metastasis.^34^ Suppression of CRC outgrowth in the liver has been attributed, in part, to cGAS/STING-dependent reprogramming of the TME, which concurrently limits the emergence of M2-skewed, tumor-supportive macrophages.^91^ Additionally, systemic delivery of synthetic STING agonists *in vivo* has demonstrated the capacity to hinder hepatic dissemination of CRC, potentially by reducing M2 polarization of TAMs.^91^ These findings imply that C5aR1 and cGAS/STING activities may be associated with TME modulation in CRC.

C5aR1 and cGAS/STING exhibit opposing regulatory roles in pathways related to radioresistance in CRC, such as Wnt/β-catenin signaling. Nuclear β-catenin enrichment in CRC correlates with greater tumor cell survival after irradiation and a higher proportion of ALDH1^+^/CD44^+^ stem-like subsets.^171^ Radioresistant SW480 cells display robust Wnt activation and predominant nuclear β-catenin, whereas HCT116 cells retain membrane-localized β-catenin and show greater radiosensitivity.^171^ In SW480 cells, the majority of DSBs were repaired, whereas they persisted in HCT116 cells 24 h after irradiation.^171^ Additionally, Wnt signaling/β-catenin positively modulates LIG4-mediated NHEJ in CRC, and blocking this pathway can increase CRC sensitivity to RT.^172^ Moreover, human CRC cells exhibit a pronounced transcriptional surge of the LIG4 gene, a pivotal ligase in the DSB repair, in apparent synchrony with aberrant β-catenin pathway hyperactivation.^172^^,^^227^ Intriguingly, endosomally localized C5a/C5aR1 orchestrates a degradation-resistant niche for β-catenin via the C5aR1/KCTD5 (potassium channel tetramerization domain containing 5) axis, streamlining its nuclear translocation and fostering tumorigenesis.^33^ In contrast, the absence of C5a or C5aR1 activity results in impaired CRC establishment due to the destabilization of β-catenin.^33^ Elevated C5aR1 transcription emerged as a molecular signature in CRC cells enriched with SET domain bifurcated histone lysine methyltransferase 1 (SETDB1), a regulator that propels tumorigenesis by modulating Wnt signaling dynamics.^43^^,^^228^ Additionally, the downstream effector IRF3 of cGAS/STING was revealed as an inhibitory modulator of Wnt signaling in CRC development, restricting β-catenin nuclear migration.^229^ These observations imply that elevated C5a/C5aR1 complement activity and impaired cGAS/STING signaling may collectively contribute to tumor progression and hinder the response to RT.

Radioresistance in CRC may result from compromised cGAS/STING signaling, potentially influenced by autophagy. Autophagy may facilitate tumor progression by inhibiting p53 activity due to genomic instability and oxidative stress. Autophagy allows tumor cells to resist separation from the basal membrane, lowering their sensitivity to therapy-induced cell death.^230^ Inhibiting autophagy induces the accumulation of cytosolic dsDNA after radiation, enhancing cGAS/STING signaling. The recruitment of Beclin-1^231^ and LC3 (microtubule-associated protein 1A/1B-light chain 3)^232^ during autophagy reflects an intrinsic mechanism that dampens overactive cGAS/STING signaling. Binding of cGAS to Beclin-1^231^ or LC3^232^ induces autophagic degradation of cytosolic dsDNA or micronuclei, impairing cGAS/STING activity. By engaging Beclin-1^231^ and driving autophagic clearance of micronuclei,^232^ the cell can suppress cGAMP generation and attenuate cGAS/STING-mediated effectors such as IFN-β. Microautophagy and p62-regulated autophagy promote STING downregulation,^233^ while protein kinase UNC-51-like kinase (ULK1) inhibits STING-dependent IRF3 activation by phosphorylating STING.^234^ Furthermore, autophagy-related genes, such as *LC3*, are highly expressed in advanced stages of CRC.^235^ Collectively, a weakened radiotherapeutic effect in CRC, reflected by impaired immune activation following DNA damage, may arise when autophagy restrains cGAS/STING signaling.

On the other hand, under low-oxygen stress, blocking C5aR1 relieves the brake on autophagy, implying that this receptor normally restrains autophagic activity in hypoxic conditions.^54^ Intense hypoxia activates the unfolded protein response, which in turn drives an accumulation of intracellular C5aR1. This increase is associated with decreased LC3-II and Beclin-1 and increased p62, suggesting a suppression of autophagy.^54^ This pattern raises the possibility that C5aR1 functionally converges with cGAS/STING signaling to govern autophagy. This interplay is unlikely to follow a straightforward pattern and instead probably involves multiple layers of regulation, warranting thorough investigation to delineate both the therapeutic upside and the potential adverse consequences of C5aR1-directed therapies. Alongside its role in autophagy, C5aR1 could intersect with other regulated death modalities, including ferroptosis,^236^ which has emerged as a compelling avenue for CRC treatment, especially in lesions that fail to respond to current standards of care.^237^ In glioblastoma models, depletion of intracellular C5aR1 via PMX205 suppresses tumor growth by enhancing ferroptotic cell death through the reduction of glutathione peroxidase 4 (GPX4) expression.^236^ Intriguingly, diminished GPX4 expression has been shown to promote lipid-peroxide-driven ferroptosis in CRC.^238^^,^^239^ Notably, ferroptosis holds considerable promise as a therapeutic strategy in CRC, in part due to its capacity to drive mitochondrial oxidative stress and lead to damage-associated molecular patterns, including mitochondrial DNA, appearing in the cytosol. These upstream signals activate the cGAS/STING/TBK1/IRF3/IFN-1 cascade, which reorganizes the tumor immune landscape by favoring M1 over M2 macrophages, enhancing CD8^+^ T cell accumulation, and reinforcing a potent IFN-1–mediated anti-tumor response.^113^^,^^201^^,^^240^ Additionally, hyperactive cGAS/STING signaling amplifies ROS, undermines GPX4 defense, and triggers lipid-peroxide-driven ferroptosis in neuronal cells.^241^ These observations point to a potential bidirectional relationship, where STING activation can enhance ferroptosis, and ferroptotic signaling may in turn amplify STING-mediated responses. In CRC, niraparib, a PARP inhibitor, strengthens RT-induced ferroptotic cell death by modulating the ATF3 (activating transcription factor)/SLC7A11 (solute carrier family 7 member 11) pathway and, in concert, activates cGAS-dependent anti-tumor immune responses.^242^ RT exerts its cytotoxic effects largely through DNA damage, a key mechanism for eliminating CRC cells. C5aR1 participates in modulating anti-tumor immunity and has emerging functions in controlling cancer cell fate. Nonetheless, whether C5aR1 shapes ferroptotic responses in CRC remains unclear. Investigating this possibility might provide a rationale for co-administering C5aR1 inhibitors with RT to capitalize on cGAS/STING-mediated programmed cell death cascades.

Taken together, cGAS/STING may serve as a downstream or co-effector in inhibiting C5aR1, thereby contributing to improved radiosensitization (Figure 2). Further studies are needed to delineate how C5aR1 inhibition enhances radiosensitization, potentially through cGAS/STING signaling. Clarifying these mechanisms could support the development of new regimens using C5aR1 blockade alone or in combination with chemotherapeutic strategies that modulate the cGAS/STING axis. Such an approach may help reduce dose-dependent toxicity while effectively targeting CRC, including immunosuppressive tumors.

## Conclusion and future perspectives

The mechanisms that allow CRC to evade treatment remain poorly understood, prompting efforts to identify their causes and develop more effective therapies for refractory disease. Emerging data offer hope for improving CRC outcomes, suggesting that RT, an affordable and feasible treatment, could provide significant benefits when combined with radiosensitizers. In animal models, C5aR1 antagonists have been shown to enhance the RT response in CRCs, including tumors with an immunosuppressive TME. Interestingly, increased C5aR1 activity inhibits STING-mediated IFN-1 production. The IRF3 signaling cascade, triggered by cGAS/STING activation and underpinned by IFN-1 as a pivotal downstream effector, serves as a critical mediator in CRC eradication post-RT. This signaling sequence can intensify anti-tumor immune responses, impair DNA repair within malignant cells, and ultimately drive their elimination. While the C5aR1 inhibitor has demonstrated improved efficacy when combined with RT in CRC, the precise mechanism underlying this effect remains unknown. Exploring how C5aR1 inhibition interacts with the cGAS/STING pathway during radiation-induced tumor clearance could uncover mechanisms of CRC resistance and unveil potential targets for future therapeutic strategies.

To clarify the mechanisms by which C5aR1-targeted therapies exert their anti-tumor effects in CRC, it is compelling to determine whether these responses may, in part, operate through the cGAS/STING pathway. Comprehensive mapping of p-TBK1, TBK1, p-IRF3, and IRF3 in CRC cells and animal models will be essential to determine how C5aR1 function aligns with cGAS/STING pathway dynamics in the context of this hypothesis. Systematic comparison of intrinsically radioresistant (HT29, SW1116, SW620, CaCo-2, DLD-1, SW480, and SW1463) with radiosensitive CRC cell lines (HCT15, HCT116, LOVO, SW48, and RKO)^223^^,^^224^ would be particularly informative, as divergent pathway activity across these panels could expose how C5aR1 intersects with cGAS/STING-mediated responses. In parallel, correlating C5aR1 expression and cGAS/STING pathway status with radiosensitivity-associated transcriptional signatures, defined by genes such as TOP2A (topoisomerase DNA II alpha), MATR3 (Matrin 3), APOL6 (apolipoprotein L6), JOSD1 (Josephin-domain-containing protein 1), and HOXC6 (Homeobox C6),^243^ and with radioresistance-linked genes, including TNFRSF13C (tumor necrosis factor receptor superfamily member 13C), CD36 (cluster of differentiation 36), ANGPTL4 (angiopoietin-like 4), LAMB3 (laminin subunit beta-3), and SERPINA1 (serpin family A member 1),^244^ may clarify how these signaling axes shape cellular radiation responses. Equally crucial is determining how the status of cGAS/STING signaling or C5aR1 expression in CRC patients aligns with clinical responses to RT, as this correlation may offer meaningful insights for guiding treatment strategies. Because anti-tumor immunity is a critical determinant of RT success, it will be essential to define how cGAS/STING activation or C5aR1 signaling levels relate to post-irradiation immune cell composition, including Tregs, M1/M2 macrophage subsets, neutrophils, CD4^+^ and CD8^+^ T cells, and DCs. A critical subsequent step is to dissect whether upregulated cGAS/STING signaling underpins the RT sensitization produced by C5aR1 inhibition across various experimental models. Accordingly, it will be essential to assess whether genetic or pharmacologic loss of cGAS, STING, or both impairs the radiosensitizing effects of C5aR1 inhibition in cell-based systems, including organoid cultures, as well as in *in vivo* CRC models. Given that stimulation of the cGAS/STING pathway is capable of inducing DNA lesions and reducing CRC cell viability, a valuable avenue of inquiry is whether pharmacologic or genetic blockade of C5aR1 can harness similar cytotoxic mechanisms, with the prospect of augmenting the efficacy of C5aR1-focused treatments in CRC.

## Acknowledgments

This work is supported by the 10.13039/100020595National Science and Technology Council, Taiwan (grant number NSTC114–2221-E-038-015).

## Author contributions

T.T.Q.P. and P.-J.L. conceptualized the review, created the figures, and wrote the manuscript. N.Q.K.L. revised and reviewed the manuscript. All authors read and approved the final manuscript.

## Declaration of interests

The authors declare no competing interests.
